# Lifestyle behaviors, metabolic disturbances, and weight gain in psychiatric inpatients treated with weight gain-associated medication

**DOI:** 10.1007/s00406-022-01442-4

**Published:** 2022-07-01

**Authors:** Maria S Simon, Barbara Barton, Anja Zagler, Katharina Engl, Leonora Rihs, Catherine Glocker, Richard Musil

**Affiliations:** Department of Psychiatry and Psychotherapy, University Hospital, LMU Munich, Nußbaumstraße 7, 80336 Munich, Germany

**Keywords:** Lifestyle, Eating behavior, Psychotropic medication, Metabolic syndrome, Weight gain, Psychiatric

## Abstract

Many psychiatric patients suffer from overweight/obesity and subsequent metabolic disturbances, where psychotropic medication is one of the main contributors. However, the magnitude of weight gain ranges individually, which leads to questioning the role of other contributors like lifestyle factors. The present study investigated several lifestyle factors among psychiatric inpatients, their relation to biological factors, and their predictive capability for weight gain during treatment. Using a naturalistic observational study design, psychiatric inpatients of all diagnoses were followed for 4 weeks from the start of treatment with weight gain-associated medication. *N* = 163 participants entered the study. Lifestyle factors were assessed by patient self-report questionnaires. Body weight change over time was calculated relative to baseline body weight. Our study provides three main findings: (1) Obesity and/or metabolic syndrome (metSy) were associated with emotional eating (disinhibition), craving for fast food and sweets, and weight cycling. (2) Patients without metSy and normal BMI experienced increased sweets craving (also for women), a more positive attitude towards drugs, and an improvement of affect (also for men). (3) Sex, presence of metSy and/or drug dosage interacted with disinhibition change, sweets craving change (trend), and fast food craving change to predict weight change over time. Furthermore, drug attitude change interacted with BMI, drug dosage, and presence of metSy to predict weight change. Lifestyle factors, especially eating behaviors, are related to metabolic disturbances and predict weight gain in interaction with clinical parameters.

**Supplementary Information:**

The online version contains supplementary material available at 10.1007/s00406-022-01442-4.

## Introduction

Previous research has investigated the role of psychotropic treatment as an important factor for weight gain and associated metabolic alterations in patients with psychiatric disorders. Relevant psychotropic drugs with a high risk for weight gain were stated: the antipsychotics clozapine, olanzapine, quetiapine, haloperidol, amisulpride, risperidone, and paliperidone, the antidepressants paroxetine, mirtazapine, doxepin, amitriptyline, and citalopram, as well as the mood stabilizers valproate and lithium [1, 2]. Subsequently, high BMI and obesity can lead to metabolic syndrome (metSy), diabetes, and cardiovascular disease [3]. Thus, serious complications arise from such disturbances as psychiatric patients show difficulties in reducing body weight [4]. Indeed, high rates of overweight, obesity, metSy, diabetes, and cardiovascular disease are well known to be associated with psychiatric diseases, and their subsequent development has been shown particularly in psychiatric patients [5–16]. However, evidence about the differential effects of disease per se and psychotropic medication is scarce. In this regard, we previously evaluated data on metabolic parameters in a transdiagnostic psychiatric sample before the start of weight gain-associated medication: 30.1% were overweight, 17.2% obese, 26.9% had metSy, 3.8% had (pre)diabetes, and 8.3% had a moderate and 1.9% a high cardiovascular risk [17]. Furthermore, Vandenberghe et al. [18] also showed that patients with psychiatric disorders (organic disorder, psychotic disorder, schizoaffective disorder, bipolar disorder, depression, and drug addiction) have high prevalence of overweight, obesity, and metSy before psychotropic treatment, already. When treated with weight gain-associated medication (clozapine, olanzapine, risperidone, quetiapine, aripiprazole, amisulpride, lithium, valproate, and mirtazapine), an early weight gain during the first 4 weeks was predictive of long-term weight gain after 12 months [18]. Interestingly, these patients did have an increase of body weight on average but also showed a broad range of weight change from weight loss in a few patients to strong weight gain [18]. These results suggest that other factors than medication contribute to a differentiation of patients who experience weight gain under medication from those who do not, next to the high occurrence of overweight and metabolic alterations even before medication onset. For example, younger age, lower initial BMI, and female sex have been discussed as potential risk factors for antipsychotic-induced weight gain [19–24].

The relevance of lifestyle factors as risk factors for weight gain has been neglected so far, but should be considered for the following reasons: Previous research has consistently shown that commonly known factors like poorer sleep quality, lower physical activity, and adverse eating behaviors are associated with physical health conditions, i.e., hypertension, dyslipidemia, cardiovascular disease, heart failure risk, metSy, type 2 diabetes mellitus, obesity, and BMI [25–33]. Furthermore, poor dietary behaviors (for example, stress-induced/emotional eating, high carbohydrate and fat intake), lacking physical activity and sedentary behavior, were especially found in patients with psychiatric disorders and associated with overweight and metSy [34–40]. Other factors like quality of sleep and life have been neglected within the context of overweight and metabolic disturbance in psychiatric patients so far. Yet, it seems that certain lifestyle factors pose a common ground in psychiatric disease and metabolic risk. However, evidence on lifestyle factors in psychiatric disorders relating to body weight gain and metabolic conditions is fragmented. Moreover, the differential impact of lifestyle factors in the context of weight gain during psychotropic therapy remains to be elucidated, completely. If relevant factors contributing to the risk of early psychotropic-induced weight gain were known, early interventions and prevention programs could target easy to assess and most of all changeable risk factors.

In this report, we first investigate lifestyle factors in psychiatric inpatients who are about to be treated with selected weight gain-associated medication to estimate the association of such factors with sex, body weight, metabolic state, and medication-associated weight gain given the body composition at medication start. Thereby, we focus on the early weight gain period of 4 weeks as this was predictive for further weight gain after [18]. We here present a variety of potential lifestyle factors and psychiatric diagnoses as previous studies are limited in this regard. While previous studies predominantly evaluated lifestyle factors and metabolic measurements in already treated patients, we here allow for a differential analysis by selecting a patient sample before the onset of weight gain-associated psychotropic medication.

## Materials and methods

### Study design

A naturalistic longitudinal observational study was performed between 2014 and 2017 at the Department of Psychiatry and Psychotherapy, University Hospital, LMU Munich, Germany. Baseline was defined by the start of weight gain-associated medication. The study was approved by the ethics committee of the LMU (protocol number 290.14) and followed the principles of good clinical practice according to the Declaration of Helsinki and its subsequent revisions. All participants provided written informed consent prior to study inclusion.

### Inclusion and exclusion criteria

Adult psychiatric inpatients (18–75 years) were included in the study if they started treatment with one of the following drugs that were associated with weight gain according to Dent et al. [2] and Bak et al. [1]: amisulpride, clozapine, haloperidol, olanzapine, paliperidone, quetiapine, risperidone, citalopram, doxepin, amitriptyline, mirtazapine, paroxetine, lithium, and valproate. All ICD-10 F-diagnoses [41] were included. Patients with severe delusions or cognitive impairments were excluded to ensure valid data from self-rated questionnaires.

### Procedure and measures

At baseline (t0) measures of metSy and BMI were taken by trained clinical personnel. Lifestyle factors were assessed by self-rating questionnaires at baseline and endpoint at week 4 (t2).

#### Biological measures

Measurement of laboratory parameters, calculation of metSy, weighing, and calculation of BMI are described elsewhere [17]. BMI was categorized according to the World Health Organization (WHO) [42]. Categories were then merged into dichotomous low/normal and overweight/obese groups. Since patients entered the study with varying baseline body weight, percentage weight change was calculated by weight gain or loss in kg from baseline to endpoint in relation to baseline body weight. To standardize the burden by medication, drug exposure was calculated as proportion of the daily maximum permissible dose or daily maximum permissible blood level at baseline, after 2 weeks, and after 4 weeks, and mean was calculated across the three time points, as well as across all drug classes, subsequently. Thus, co-medication and different drug-dependent therapeutic dosage proportions are taken into account, and a standardized potential maximum drug exposure was established as reference value (100% drug exposure by all 14 drugs).

#### Behavioral measures

Eating behavior was assessed using the three-factor-eating questionnaire (TFEQ [43]) which measures cognitive control of eating behavior (dietary restraint), the interference of eating behavior (disinhibition), and the experienced feeling of hunger. The German version Fragebogen zum Essverhalten (FEV) was validated by Pudel and Westenhöfer [44]. Sum scores were added up for each scale with higher score indicating higher expression of habits.

Food craving was assessed by rating how often 28 different foods listed were craved for during the last month (Food-Craving Inventory [45], German version by Dalkner et al., personal communication). Higher values of the total sum score indicate higher craving. The subscale sum scores fast food craving, carbohydrate craving, sweets craving, and fat craving were calculated, separately.

Weight cycling was assessed according to Field et al. [46] by at least 3 times a loss of at least 9 kg (severe) or a loss of at least 4.5 kg (mild). No weight cycling was coded, if weight loss was less than 4.5 kg and/or less than 3 times. The German version was adapted and enhanced (Wallner-Liebmann, personal communication) in which weight loss did not necessarily have to be intentional.

Physical activity during the past 7 days was measured using the international physical activity questionnaire (IPAQ long version [47], German long version validated by Hagströmer et al. [48]). For our study purposes, the domain “time spent sitting” was used to indicate sedentary behavior of patients.

Of the Lancashire quality-of-life profile (LQLP) [49], two scales were used: The affect balance scale (ABS [50, 51] assesses the health-related life quality and subjective well-being. The sum score of negative affect items is subtracted from the sum score of positive affect items and represents the affect balance. The Rosenberg Self-Esteem Scale assesses a self-esteem sum score with higher score indicating higher self-esteem [52].

Subjective sleep quality over the past 4 weeks was measured using the Pittsburgh Sleep Quality Index (PSQI) [53]. The total score was categorized into good (score below 6) or poor (score above 5) sleep quality, or chronic (score above 10) sleep disturbances. The German version was developed by Riemann and Backhaus [54] as a 2-week version and was used assessing the past 4 weeks for our purposes.

The drug attitude inventory (DAI; original version by Hogan et al. [55], 10-item version by Nielsen et al. [56]) was used to assess the subjective perception of psychotropics, indirectly reflecting compliance. A sum score below 0 indicates a negative attitude, above 0 a positive attitude.

### Statistical analyses

All calculations were done using SPSS Statistics 25. Tests were conducted two-sided and the significance threshold was set at *α* = 0.05. Lifestyle factors were analyzed regarding their baseline levels and change over time which were calculated subtracting t0 from t2. Thus, a positive score represents an increase and a negative score represents a reduction. Descriptive statistics are presented by frequency, mean and standard deviation, or median and interquartile range where applicable. To compare baseline and endpoint values, paired *t* test was carried out for continuous variables. In case of inhomogeneous variances, Welch test was used. Chi-square test was applied for categorical data. For correlation coefficients, Spearman rank correlation was used. Lifestyle factors at baseline were further contrasted by the dichotomized variables sex, BMI (overweight/obese ≥ 25 kg/m^2^), and presence of metSy. To test for significance, Student’s *t* test was used for continuous data and Chi-square test for categorical data. Change of lifestyle factors was tested among these subgroups (women, men, low/normal BMI, overweight/obesity, no metSy, and presence of metSy) using paired *t* test. Multiple test correction was dispensed due to the exploratory hypothesis-generating character of the study and partly small sample sizes in subgroups. Multiple regression analyses were performed to explore the predictive value of lifestyle factors at baseline and their change from baseline to endpoint for the change of body weight over the treatment period. BMI, sex, presence of metSy, drug dosage, and their two-way interaction with each lifestyle factor were also included in the model.

## Results

### Demographics

The total sample of eligible patients at baseline consisted of 163 (60.1% (*n* = 98) men and 39.9% (*n* = 65) women) psychiatric inpatients. *N* = 98 patients participated in the follow-up assessment at week 4, of which *n* = 52 patients filled out any questionnaires. Demographical and clinical characteristics of the study sample are displayed in Table 1. Noteworthy, the mean percent drug dosage had a high range from 0.05 to 18.6%, where the upper quartile was at 5.3% indicating higher values. Further information on demographical data and clinical characteristics can be found in Barton et al. [17].

**Table 1:** Demographical and clinical characteristics of the study sample

	*M*	SD	*N*
Age at study entry (years)	39.82	15.09	163
BMI (kg/m^2^)	25.53	5.44	163
Weight change t0–t2 (%)	2.24	4.23	104
Drug dosage^a^ (%)	3.8	4.19	163
Age of onset of psychiatric symptomatology (years)	29.2	15.47	161
Duration of psychiatric disease history (years)	10.74	11.14	161
	%		*N*
Overweight/obese BMI >24	47.2		77
Low/normal BMI <25	52.8		86
Metabolic syndrome yes	29.7		46
Drugs			
Quetiapine	29.4		48
Mirtazapine	20.9		34
Risperidone	12.9		21
Lithium	6.7		11
Valproate	6.1		10
Olanzapine	5.5		9
Amisulpride	5.5		9
Clozapine	3.7		6
Citalopram	3.1		5
Doxepin	3.1		5
Haloperidol	1.2		2
Amitriptyline	1.2		2
Paroxetine	0.6		1
Diagnoses^b^			
Depression	46		75
Bipolar disorders	20.9		34
Drug addiction	20.2		33
Neurotic/stress-related/somatoform disorders	12.3		20
Psychotic disorders	11.0		18
Personality disorders	8.6		14
Schizoaffective disorders	5.5		9
Organic disorders	2.5		4
Persistent/other/unspecified affective disorders	1.2		2
Developmental disorders	0.6		1

^a^Proportion of maximum permissible dose or blood level across treatment period.

^b^The cumulative percentage exceeds 100% due to comorbidity.

*M* mean; SD standard deviation; *n* number of participants.

### Lifestyle characteristics at baseline and their change over time

Eating habits, food craving, sitting time, and self-esteem did not change from baseline to 4 weeks of treatment. There was a significant increase of scores for attitude towards drugs to a more positive attitude and for affect balance score to a less negative affect, as well as a trend for sweets craving (more craving after treatment). A significant decrease emerged for total sleep quality score towards a less-disturbed sleep quality. Results are displayed in Table 2.

**Table 2:** Lifestyle characteristics of psychiatric inpatients across diagnoses before and after treatment

	t0	*n*	t2	*n*	test (SD)	*n*	*P*	CI
FEV								
Cognitive control *M* (SD)	7.19 (4.72)	104	6.22 (5.62)	18	*t* = 0.49 (4.21)	15	0.631	[− 1.80; 2.86]
Disinhibition *M* (SD)	6.00 (3.74)	103	6.70 (3.94)	20	*Z* = − 0.25	17	0.805	–
Hunger *M* (SD)	5.20 (3.67)	109	6.94(4.28)	17	*t* = 1.09 (3.55)	15	0.293	[− 0.96; 2.96]
Food craving								
Total score *M* (SD)	30.87 (15.73)	107	32.21 (16.22)	47	*t* = − 1.16 (17.23)	45	0.253	[− 8.15; 2.20]
Fat *M* (SD)	7.17 (5.49)	115	7.94 (5.67)	49	*t* = − 0.93 (4.97)	48	0.357	[− 2.11; 0.78]
Sweets *M* (SD)	9.94 (6.52)	112	10.64 (6.56)	50	*t* = − 1.68 (6.87)	46	0.101^+^	[− 3.73; 0.34]
Carbohydrate *M* (SD)	8.45 (5.74)	114	8.70 (5.20)	50	*t* = − 0.28 (6.13)	49	0.781	[− 2.01; 1.52]
Fast food *M* (SD)	5.06 (3.66)	116	5.61 (3.58)	51	*t* = − 1.17 (3.49)	50	0.246	[− 1.57; 0.41]
Weight cycling								
No (%)	65.10	71	–	–	–	–	–	–
Mild (%)	29.40	32	–	–	–	–	–	–
Severe (%)	5.50	6	–	–	–	–	–	–
Sitting time								
Minutes per day *M* (SD)	372.88 (202.88)	106	363.99 (194.91)	48	*t* = − 0.14 (197.17)	46	0.886	[− 62.74; 54.36]
Life quality								
ABS *M* (SD)	− 1.58 (2.44)	115	− 1.13 (2.45)	52	*t* = − 3.09 (2.32)	51	0.003*	[− 1.65; − 0.35]
Self-esteem *M* (SD)	3.49 (2.63)	114	3.88 (2.73)	52	*t* = − 0.92 (2.31)	50	0.362	[− 0.96; 0.36]
Sleep quality								
Total score *M* (SD)	10.32 (3.80)	78	9.97 (3.03)	29	*t* = 2.56 (1.58)	22	0.018*	[0.16; 1.57]
Good (%)	11.50	9	6.90	2	*χ*^*2*^ = 0.73		0.693	–
Poor (%)	41.00	32	48.30	14				
Chronic (%)	47.40	37	44.80	13				
Attitude towards drugs								
Total score *M* (SD)	1.46 (4.21)	108	3.28 (3.94)	47	*t* = − 2.35 (4.47)	44	0.023*	[− 3.12; − 0.24]

ABS affect balance scale; *M* mean; SD standard deviation; CI 95% confidence interval; ^+^*p* ≤ 0.10, *p ≤ 0.05.

### Association of lifestyle change over time with drug dosage

To examine the potential role of medication for the observed changes (see Sect. Lifestyle characteristics at baseline and their change over time), the association with drug dosage was investigated. Change of self-esteem over time was positively correlated with drug dosage at the border of significance (*n* = 50; Spearman-Rho = 0.27; *p* = 0.058): the higher the dosage, the higher the improvement of self-esteem. No other lifestyle changes were associated with drug dosage.

### The role of sex, BMI, and presence of metabolic syndrome for baseline lifestyle factors and their change over time

*FEV* Regarding eating behavior, women showed higher cognitive control than men, and overweight/obese patients and patients with metSy showed higher disinhibition and hunger compared to patients with normal BMI and without metSy (Table 3 ).

**Table 3:** Lifestyle characteristics with regard to sex, BMI, and presence of metabolic syndrome: eating habits, weight cycling, and sedentary behavior

	MD (SE)	test	*p*	CI
FEV				
Cognitive control				
Sex	2.70 (0.90)	*t* = 2.99	0.003*	[0.91; 4.49]
BMI	− 1.11 (0.92)	*t* = − 1.20	0.233	[− 2.94; 0.72]
MetSy	0.65 (1.07)	*t* = 0.61	0.541	[− 1.46; 2.77]
Disinhibition				
Sex	1.18 (0.74)	*t* = 1.61	0.111	[− 0.28; 2.65]
BMI	− 2.53 (0.70)	*t* = − 3.63	< 0.001*	[− 3.91; − 1.15]
MetSy	− 2.25 (0.80)	*t* = − 2.83	0.006*	[− 3.83; − 0.67]
Hunger				
Sex	− 0.47 (0.69)	*t* = − 0.68^a^	0.496	[− 1.84; 0.90]
BMI	− 1.39 (0.69)	*t* = − 2.01	0.047*	[− 2.77; − 0.02]
MetSy	− 1.55 (0.78)	*t* = − 1.98	0.051*	[− 3.10; < 0.01]
Food craving				
Total score				
Sex	− 2.09 (3.10)	*t* = − 0.68	0.501	[− 8.24; 4.05]
BMI	− 5.31 (3.01)	*t* = − 1.76	0.081^+^	[− 3.42; 0.63]
MetSy	− 5.34 (3.53)	*t* = − 1.51	0.134	[− 12.35; 1.67]
Fat				
Sex	− 3.04 (1.00)	*t* = − 3.04	0.003*	[− 5.02; − 1.06]
BMI	− 1.39 (1.02)	*t* = − 1.37	0.175	[− 3.42; 0.63]
MetSy	− 1.31 (1.25)	*t* = − 1.04^a^	0.302	[− 3.83; 1.21]
Sweets				
Sex	0.36 (1.26)	*t* = 0.29	0.773	[− 2.13; 2.86]
BMI	− 2.23 (1.22)	*t* = − 1.83	0.070^+^	[− 4.65; 0.19]
MetSy	− 2.93 (1.40)	*t* = − 2.08	0.040*	[− 5.71; − 0.14]
Carbohydrate				
Sex	1.16 (1.09)	*t* = 1.06	0.291	[− 1.00; 3.32]
BMI	− 0.04 (1.08)	*t* = − 0.03	0.973	[− 2.18; 2.10]
MetSy	0.20 (1.18)	*t* = 0.17	0.867	[− 2.14; 2.53]
Fast food				
Sex	− 1.02 (0.68)	*t* = − 1.50	0.137	[− 2.38; 0.33]
BMI	− 1.29 (0.67)	*t* = − 1.92	0.057*	[− 2.62; 0.04]
MetSy	-0.17 (0.77)	*t* = − 0.23	0.822	[− 1.70; 1.36]
Weight cycling				
Sex		*χ*^*2*^ = 2.13	0.345	
BMI		*χ*^*2*^ = 4.30	0.117	
MetSy		*χ*^*2*^ = 10.42	0.005*	
Sitting time				
Sex	6.02 (40.05)	*t* = 0.15	0.881	[− 73.41; 85.45]
BMI	22.64 (39.54)	*t* = 0.57	0.568	[− 55.76; 101.05]
MetSy	− 64.97 (45.74)	*t* = − 1.42	0.159	[− 155.73; 25.79]

Sex comparison of women vs men; BMI comparison low-normal vs obese-adipose; MetSy metabolic syndrome according to International Diabetes Federation (IDF, see [17]) yes vs no; MD mean difference; SE standard error of mean difference; CI 95% confidence interval; ^a^Welch test; ^+^*p* ≤ 0.10, *p ≤ 0.05.

*Food craving* Men had higher fat craving than women. Overweight/obese patients had higher fast food craving, as well as higher sweets and total food craving as a trend (Table 3). Furthermore, patients with metSy showed higher sweets craving as well as a more severe weight cycling profile (Table 3). Women, patients with normal BMI, and patients without metSy showed a significant increase of sweets craving over time (Table 5).

*ABS and self-esteem* Negative–positive affect balance in men changed significantly to a less negative affective state over time, as well as in patients with normal BMI, and patients without metSy. Self-esteem in men significantly increased over time (Table 5).

*Sleep quality* Concerning sleep, women had a more severely disturbed sleep profile compared to men and sleep quality score was higher (more disturbed) in patients without metSy (Table 4). In men, sleep quality significantly improved over time, as well as in patients with normal BMI and patients with metSy (Table 5).

**Table 4:** Lifestyle characteristics with regard to sex, BMI, and presence of metabolic syndrome: life quality, sleep quality, and drug attitude

	MD (SE)	test	*P*	CI
Life quality				
ABS				
Sex	−0.62 (0.46)	*t* = −1.35	0.178	[−1.53; 0.29]
BMI	−0.22 (0.46)	*t* = −0.49	0.628	[−1.13; 0.68]
MetSy	0.62 (0.51)	*t* = 1.23	0.223	[−0.39; 1.62]
Self-esteem				
Sex	−0.29 (0.50)	*t* = −0.58	0.561	[−1.29; 0.70]
BMI	−0.39 (0.49)	*t* = −0.78	0.435	[−1.37; 0.59]
MetSy	0.64 (0.56)	*t* = 1.15	0.253	[−0.47; 1.75]
Sleep quality				
Total score				
Sex	2.17 (0.88)	*t* = 2.46	0.016*	[0.41; 3.93]
BMI	0.81 (0.86)	*t* = 0.94	0.348	[−0.90; 2.53]
MetSy	−2.25 (0.96)	*t* = −2.35	0.022*	[−4.16; −0.34]
Categories				
Sex		*χ*^*2*^ = 7.80	0.020*	
BMI		*χ*^*2*^ = 1.86	0.394	
MetSy		*χ*^*2*^ = 4.01	0.135	
Attitude towards drugs				
Sex	−0.98 (0.81)	*t* = −1.21	0.230	[−2.60; 0.63]
BMI	1.39 (0.80)	*t* = 1.73	0.087^+^	[−0.20; 2.98]
MetSy	1.53 (0.87)	*t* = 1.75	0.083^+^	[−0.20; 3.27]

Sex comparison women vs men; BMI comparison low-normal vs obese-adipose; MetSy metabolic syndrome according to IDF [17] yes vs no; ABS affect balance scale; *MD* mean difference; *SE* standard error of mean difference; *CI* 95% confidence interval; ^+^*p* ≤ 0.10, *p ≤ 0.05.

**Table 5:** Significant change of lifestyle factors over time in several patient subgroups

	*M*	*t*	*p*	CI
Sweets craving				
Women	3.80	2.49	0.022*	[0.61; 6.99]
Normal BMI	3.67	2.75	0.011*	[0.91; 6.42]
No metSy	2.41	2.12	0.042*	[0.09; 4.72]
ABS				
Men	1.26	3.19	0.004*	[0.45; 2.07]
Normal BMI	1.52	3.10	0.005*	[0.51; 2.53]
No metSy	1.23	3.27	0.002*	[0.46; 1.99]
Self-esteem				
Men	0.96	2.16	0.040*	[0.05;1.88]
Sleep quality				
Men	− 0.86	− 2.48	0.028*	[− 1.60; − 0.11]
Normal BMI	− 1.00	− 2.31	0.038*	[− 1.93; − 0.07]
MetSy	− 0.83	− 2.05	0.056^+^	[− 1.69; 0.02]
Attitude towards drugs				
Normal BMI	3.00	2.33	0.031*	[0.31;5.69]
No metSy	2.13	2.36	0.025*	[0.28;3.98]

MetSy metabolic syndrome according to IDF [17]; ABS affect balance scale; *M* mean; CI 95% confidence interval; ^+^*p* ≤ 0.10, *p ≤ 0.05.

*Drug attitude* Interestingly, a trend for a more positive attitude towards drugs emerged in overweight/obese patients and patients with metSy (Table 4). Drug attitude changed to a more positive evaluation in patients with normal BMI and without metSy (Table 5).

### The relation between lifestyle factors and weight gain after treatment under covariate consideration

Based on the previous sections, potential relevant covariates were included to predict weight gain by the expression of lifestyle factors.

*Baseline lifestyle factors* Multiple regression analyses revealed a significant model for experience of hunger at baseline (*R*^2^ = 0.26; *F*(9, 62) = 2.37; *p* = 0.023), but none of the coefficients reached significance. No other significances emerged for baseline lifestyle factors on weight change over time.

*Lifestyle factor change* The overall model for disinhibition was significant (*R*^2^ = 0.90; *F*(9, 7) = 6.60; *p* = 0.011), while disinhibition change was predictive for weight change independently of covariates, as well as was BMI. Furthermore, disinhibition change interacted with and sex, drug dosage, and the presence of metSy to predict weight change (Table 6, Supplementary table 1, and Supplementary figs. 1 and 2).

**Table 6:** Significant predictors of weight change over time

	*ß*	*t*	df	*p*	CI
FEV—disinhibition					
Disinhibition change	4.12	2.80	7	0.027*	[0.65; 7.68]
Disinhibition change × sex	−1.63	−2.81	7	0.026*	[−3.18; −0.27]
Disinhibition change × drug dosage	−1.25	−2.61	7	0.035*	[−0.35; −0.02]
Disinhibition change × metSy	−1.19	−2.70	7	0.031*	[−4.36; −0.29]
Food craving—sweets					
Sweets craving change × sex	0.51	1.94	36	0.061^+^	[−0.02; 0.72]
Sweets craving change × drug dosage	0.56	1.96	36	0.057^+^	[− 0.002; 0.12]
Sweets craving change × metSy	−0.76	−1.88	36	0.068^+^	[−1.26; 0.05]
Food craving—fast food					
Fast food craving change × drug dosage	0.71	2.96	40	0.005*	[0.04; 0.19]
Food craving total score					
Food craving × drug dosage	0.57	2.13	35	0.040*	[0.001; 0.05]
Attitude towards drugs					
Drug attitude change	3.48	3.35	34	0.002*	[0.79; 3.23]
Drug attitude change × BMI	−3.73	−3.19	34	0.003*	[−0.13; −0.03]
Drug attitude change × drug dosage	−0.85	−3.10	34	0.004*	[−0.14; −0.03]
Drug attitude change × metSy	1.15	2.72	34	0.010*	[0.34; 2.32]

metSy metabolic syndrome according to IDF [17]; × multiplicative operator indicating the interaction term; df degrees of freedom; CI 95% confidence interval; ^+^*p* ≤ 0.10, *p ≤ 0.05.

Regarding the predictive values of food craving change over time for weight change, some trends emerged for single coefficients (but not for the overall model): sweets craving change interacted with sex, drug dosage, and presence of metSy to predict weight change (Table 6, Supplementary table 1 and Supplementary figs. 3 and 4). Furthermore, a strong trend emerged for the overall model of fast food craving change over time (*R*^2^ = 0.32; *F*(9, 40) = 2.06; *p* = 0.058), while fast food craving change interacted with drug dosage to predict weight change (Table 6 and Supplementary table 1). The total food craving change score interacted with drug dosage to predict weight change (Table 6). The overall model for change of drug attitude was not significant, while change of drug attitude significantly predicted weight change (Table 6). Furthermore, drug attitude change interacted with BMI, drug dosage, and presence of metSy to predict weight change (Table 6).

## Discussion

In the present study, we investigated the presence of lifestyle factors before and after treatment with weight gain-associated medication and in relation to BMI, metSy, sex, dosage of drugs, and change of body weight over time. At baseline, nearly half of the patients were overweight/obese, almost one-third showed presence of metSy, and patients gained 2.24% of baseline body weight over the treatment period. This is in line with the previous reports of higher occurrence of obesity and metabolic dysbalances in psychiatric patients [16, 34].

### Clinical patient characteristics are differently associated with lifestyle factors

*FEV* Eating habits were moderately expressed with regard to norm standards [44], but differed at baseline due to patient characteristics: women showed higher cognitive control and overweight/obese patients and patients with metSy showed especially higher disinhibition (emotional eating) and hunger. Thus, disturbed eating behavior is clearly associated with already present metabolic abnormalities. Prior research confirms that women show higher restriction (cognitive control), but also disinhibition/emotional eating and hunger at the same time [57, 58]. Furthermore, disinhibition and hunger were associated with higher BMI [58, 59]. Sentissi et al. [58] also found a trend for higher disinhibition/hunger and lower restraint (cognitive control) in schizophrenic patients with compared to without metSy. In our study, patients were investigated transdiagnostically and treated with weight gain-associated medication. In line, disinhibition trended for a higher expression under atypical antipsychotics than conventional neuroleptics [58]. As a potential effect of diagnosis or symptoms, individuals with mood disorders showed more cognitive restraint and disinhibition concerning food intake compared to population norms [59], and anhedonia was particularly associated with emotional/uncontrolled eating (disinhibition) [60]. Thus, investigating a drug- and symptom-specific effect for certain eating behaviors in future analyses may be worthwhile to refine population characteristics that can be targeted with interventions on a behavioral basis.

*Food craving* Men showed higher fat craving, and overweight/obese patients and patients with metSy showed higher fast food craving and sweets craving, respectively. Again, already existing metabolic abnormalities are clearly associated with craving for unhealthy food. In a mixed sample from healthy and psychiatric ill individuals, women craved simple sugars/trans fats more than men, while men craved fast food (saturated fats/high calorie content) more than women [61]. In exploratory analyses of a sample of college students, obese individuals were more likely craving high fats than normal weight participants and high-fat scores were positively associated with BMI [62]. Furthermore, in line with our results, patients with severe mental illness and overweight/obesity showed higher craving for fast food fats [63]. The overlap between fat and fast food craving is difficult to quantify, though possibly considerably large. In this light, our results seem to along with the presented findings from literature.

*Weight cycling* Presence of metSy was also associated with more severe weight cycling which confirms previous results of higher cardiometabolic risk in weight cyclers [64].

*Sleep quality* Most patients (88.4%) were poor or chronically disturbed sleepers and sleep quality was more disturbed in women and in patients without metSy. Poor sleep quality has also been found in a psychiatric outpatient sample with a very similar rate of 87.4% and sleep quality was lower in psychiatric in- and out-patients compared to controls [65, 66]. So far, previous studies had not related quality of sleep with metabolic disturbances in psychiatric patients.

*Sitting time* A study by Alswat et al. [34] demonstrated an association of sedentary behavior with metabolic disturbances: psychiatric inpatients with already existent metSy more likely reported sedentary lifestyle, had higher BMI, hypertension, hyperlipidemia, and diabetes compared to patients without metSy. On the contrary, we did not find such association.

### Clinical patient characteristics are differently associated with the change of lifestyle factors during psychotropic treatment

Over time, attitude towards drugs, sleep quality, and negative affect improved, indicating an improvement of psychiatric treatment targets, while craving for sweets marginally deteriorated in the total patient group. Of all lifestyle factors, only improvement of self-esteem strongly trended for a positive association with drug dosage. In a schizophrenic sample, patients associated changes in dietary habits like increase in high carbohydrate and fat intake with medication use [39]. However, this study only assessed the subjective perception of patients of this relationship in a cross-sectional design.

*Food craving* Kazes et al. [67] found suddenly increased appetite for sweets and fatty food before weight gain after antidepressant onset. In our study, other factors seem to play a role for the changes of lifestyle behaviors during the treatment period: Patients without metSy and normal BMI, as well as women, showed increased sweets craving over time. This leads to the conclusion that patients without metabolic abnormalities experience a deterioration of unhealthy sweets craving over time as opposed to patients with presence of those abnormalities who already show such craving at baseline (see Sect. Clinical patient characteristics are differently associated with lifestyle factors). Consequently, it can be suspected that the increase and intensity of appetite may reach a plateau at some point even if medication is still taken. One study found no changes over time across different food craving types after treatment, but patients with normal weight presented an increase of the complex carbohydrates/proteins craving score [63]. This study also found a change of fast food fats craving during treatment with clozapine which was associated with male sex and baseline BMI [63]. The limitation to the use of only one antipsychotic drug in this study poses a substantial difference to our multi-drug approach.

*ABS and self-esteem* Patients without metSy and normal BMI, as well as men showed an improvement of affect and men felt more self-esteem over time.

*Sleep quality* Sleep quality improved in patients with metSy, normal BMI, and in men.

*Drug attitude* Patients without metSy and normal BMI gained a more positive attitude towards drugs.

These findings suggest that patients without metabolic abnormalities are more capable of experiencing improvement of mood and positive drug attitude after treatment. Thus, it is tempting to speculate that patients with such abnormalities might be less responsive to treatment, or experience less positive affect, or are less treatment compliant due to higher metabolic burden.

### Weight gain during psychotropic treatment is mainly predicted by changing eating habits and drug attitude in interaction with drug dosage, metabolic disturbances, and sex

*FEV* Predictive of weight change during treatment was the change of disinhibition and the interaction of disinhibition change with sex, drug dosage, and presence of metSy. Though change of disinhibition itself over time was not significant per se, a subliminal effect in interaction with other factors was sufficient to indicate weight gain.

*Food craving* The interaction of fast food craving change with drug dosage showed a strong predictive effect, but the non-parametric correlation was weak. It is arguable whether especially low and high values can be considered as expectable values or as outliers, which complicates the decision for parametric or non-parametric test approach. Here, we decided to include such values in the parametric and non-parametric analyses as they seem clinically reasonable and reach high values in the interaction term due to its multiplicative nature. Like with disinhibition, the craving change itself over time per se was not present, but a subliminal effect in interaction with drug dosage was sufficient to indicated weight gain. The interaction of sweets craving change with sex, drug dosage, and presence of metSy showed a considerable trend. Sweets craving deteriorated over time and its change interacted with relevant variables. Like with disinhibition, the presence of metSy moderated the relationship of eating behavior and weight change, i.e., absent metabolic disturbances resulted in weight gain under deterioration of eating habits and vice versa in presence of metabolic disturbances. This may indicate the higher capacities for metabolic and eating deteriorations among initially unaffected patients, while present disturbances may not lead to further measurable deterioration over such short time. Previously, Garriga et al. [63] found increases in fast food craving to be predictive for weight gain after clozapine treatment in patients with severe mental illness. In another study, the food craving total score as well as the preoccupation with food and loss of control subscales at baseline were significantly associated with weight gain after antipsychotic intake only in females [57]. To our knowledge, these and our results are the first to provide insights in potential targets for behavioral monitoring and interventions, but more research is needed.

*Drug attitude* Strong predictive effects were found for change of drug attitude and the interaction of drug attitude change with BMI, drug dosage, and presence of metSy. Thus, drug attitude as an indicator of treatment compliance should be taken into account when investigating the significant interactions with drug dosage, and presence of metSy also stood out to predict weight gain after treatment period.

Many widely used psychotropic drugs are associated with weight gain, but effective treatment alternatives are lacking. Therefore, it is desirable to investigate potential risk factors for such weight gain to improve the management of unwanted side effects. The here-presented results serve as hypothesis-generating insights that should be verified in future studies and build the grounds for the development of interventions to combat weight gain.

### Limitations

Several limitations need to be considered. First, the study included a follow-up time of only 4 weeks. This approach was based on previous literature pointing to a special relevance of early weight gain during the first 4 weeks of treatment for continuing weight gain after [18]. Thus, determining risk for early weight gain is of special interest to deduce early intervention measures. Nevertheless, not being able to reproduce the long-term effects found in the literature poses a shortcoming of this study. Interestingly, even though the first weeks of treatment are often affected by dosage finding, the dose seems less relevant for metabolic effects in most cases [68]. Prior medication at any time before the study was not taken into account. Even though the patients entered the study drug-free regarding weight gain-associated medication, they likely were not drug-naive. This may have influenced the baseline body weight already and metabolic alterations may be long-lasting processes which complicates an estimation of the possible impact of previous medication intake throughout life. Thus, the results may be biased towards the relationship of lifestyle variables with weight gain attributable to the medication intake. However, following a naturalistic approach, this patient collective represents the clinical situation where patients have likely been treated before weight gain-associated medication may be given regardless of the BMI. Thus, baseline BMI was considered in the analyses to estimate weight gain and possible further weight gain. The results present some consistency. However, the overall picture is somewhat scattered. Due to the unexpectedly large number of drop-outs during the study, the application of an imputation method was dispensed. The reasons for drop-out were not registered systematically. Main reasons were lost to follow up due to discharge from the clinic and lacking patient motivation. Furthermore, the FEV as was added as a follow-up measure during the course of the study explaining the small amount of available values. It is debatable whether non-significant findings are a result of type II error, i.e., lack of statistical power, or of truly non-existent associations. Therefore, such findings should not be generalized to the target population, but may serve as indicators for aspects to be replicated in future studies. In this light, the almost significant trends can rather be interpreted as a positive finding. Generally, the present results should be viewed as preliminary due to potential uncontrolled bias. In future, verification in a bigger sample is needed to see whether a more elaborate pattern emerges or whether these single parameters that showed significant associations should be selected, specifically. Regarding the operationalization of eating behavior, the FEV has to be criticized for its high correlation of the disinhibition and hunger scale, i.e., their limited discriminability [44]. Furthermore, no actual measurement of food intake was carried out, while craving and eating behavior are only indirect surrogates of the actual amount of food eaten. Thus, it is difficult to attribute weight gain to caloric intake. On the other hand, using such scales provides clearer indications for behavioral intervention targets. Finally, other factors beyond the presented clinical and lifestyle characteristics should be considered in future studies. Since a relationship of factors like childhood trauma and socioeconomic variables with weight gain was established, those parameters should be taken into account [69, 70]. In the present study, socioeconomic factors were measured, but their evaluation was disregarded due to questionable validity of the instrument. A currently running project investigates the effects of both aspects for weight gain.

## Conclusion

Overall, certain dysfunctional eating behaviors and their development over time seem to be related to metabolic disturbances in psychiatric patients. In particular, in the presence of metabolic disturbances, patients showed dysfunctional eating at baseline, while eating behavior worsened over time when metabolic disturbances were not present. Sex seems to be related differentially. Men and patients without metSy were particularly vulnerable to develop weight gain when disinhibition (emotional eating) increased during treatment. Also, the combined effect of emotional eating deterioration and medication burden predicted weight gain. Likewise, women and patients without metSy were particularly vulnerable to develop weight gain when sweets craving increased during treatment. The combined effect of sweets craving deterioration and medication burden was also predictive of weight gain. Finally, the combined effect of fast food craving deterioration and medication burden seems predictive of weight gain. Thus, psychotropic drug consumption, metabolic condition, and sex are important variables to consider when changing eating behaviors are monitored and targeted to predict and prevent weight gain during psychotropic therapy. A monitoring strategy of changing eating habits, especially in vulnerable subgroups, should be implemented accompanying body weight measures throughout treatment.

## Supplementary Information

Below is the link to the electronic supplementary material.

Supplementary file1 (DOCX 152 KB)

## Data Availability

Data are available upon request.

## Code availability

The SPSS script is available from the first author upon request.
