# Supplementary material for: Lifestyle behaviors, metabolic disturbances, and weight gain in psychiatric inpatients treated with weight gain-associated medication
Source: Eur Arch Psychiatry Clin Neurosci. 2022 Jul 1;273(4):839–51. doi: 10.1007/s00406-022-01442-4 (PMC10238335; doi:10.1007/s00406-022-01442-4)
Supplement: Supplementary file 1 — Supplementary file1 (DOCX 152 KB) [file 406_2022_1442_MOESM1_ESM.docx]

**Supplementary material**

Supplementary table 1. Correlations of eating behavior by drug dosage interaction terms with weight change.

|  | *Pearson r* | *p* | *Spearman-Rho* | *p* |
| --- | --- | --- | --- | --- |
| disinhibition change*drug dosage  (N=17) | 0.35 | 0.16 | 0.51 | 0.04* |
| fastfood craving change*drug dosage  (N=50) | 0.40 | 0.004* | 0.09 | 0.54 |
| sweets craving change*drug dosage  (N=46) | 0.19 | 0.20 | 0.20 | 0.19 |

Notes. The interaction of disinhibition change and drug dosage positively correlates to weight gain using Spearman rank correlation. Further, the interaction term sweets craving change-drug dosage was positively, though only descriptively, associated with increase of weight. Notably, the interaction term only correlated weakly though positively with weight change using Spearman rank correlation. ^*^*p* ≤ .05


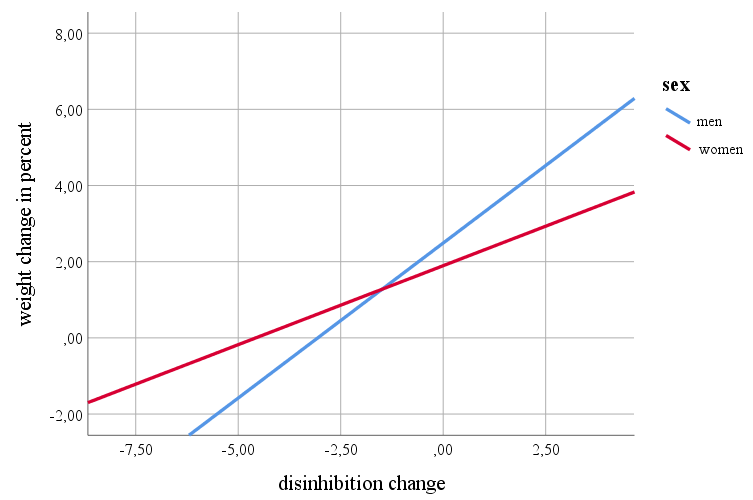
Supplementary figure 1. Relationship of disinhibition change and weight change in women and men.

Notes. Men showed a greater increase of weight with increase of disinhibition than women. Change of parameters was calculated after four weeks of treatment. Positive values indicate an increase in body weight and emotional eating, negative values indicate a decrease.

Supplementary figure 2. Relationship of disinhibition change and weight change in patients with and without metabolic syndrome.


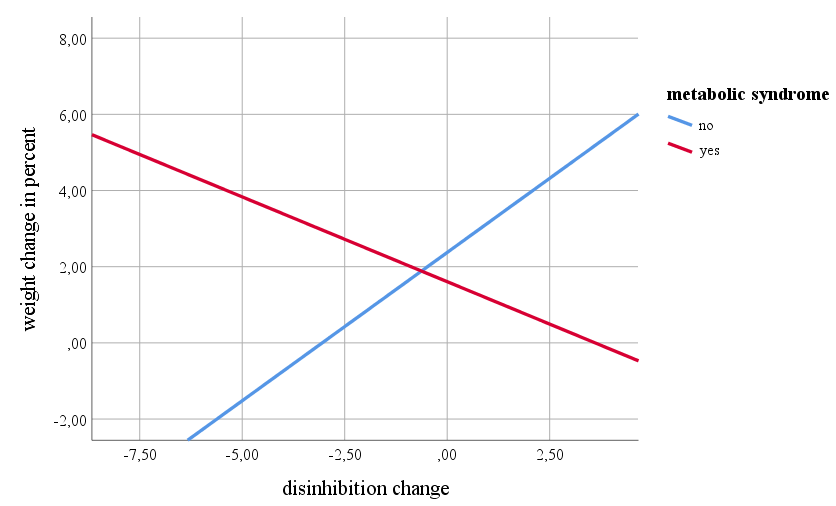


Notes. With increasing disinhibition, patients without metabolic syndrome showed an increase of weight while patients with metabolic syndrome showed a decline of weight. Change of parameters was calculated after four weeks of treatment. Positive values indicate an increase in body weight and emotional eating, negative values indicate a decrease. Metabolic syndrome was defined according to IDF (see [17]) at baseline.

Supplementary figure 3. Relationship of sweets craving change and weight change in women and men.


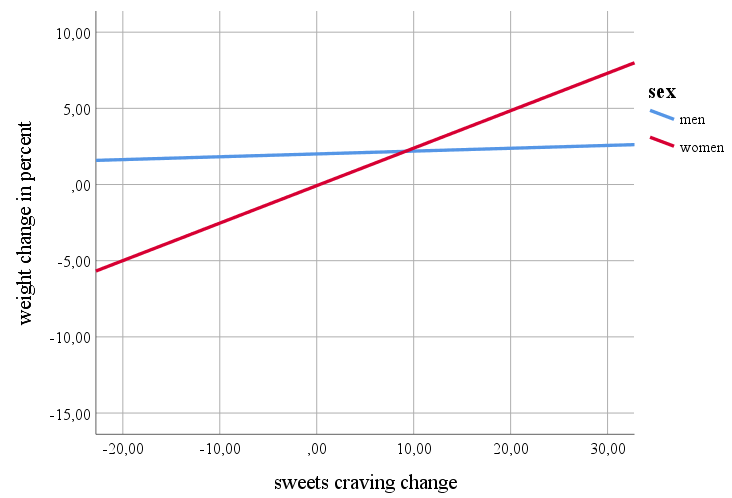


Notes. Women showed a greater increase of weight with increase of sweets craving than men who showed almost no change at all. Change of parameters was calculated after four weeks of treatment. Positive values indicate an increase in body weight and sweets craving, negative values indicate a decrease.

Supplementary figure 4. Relationship of sweets craving change and weight change in patients with and without metabolic syndrome.


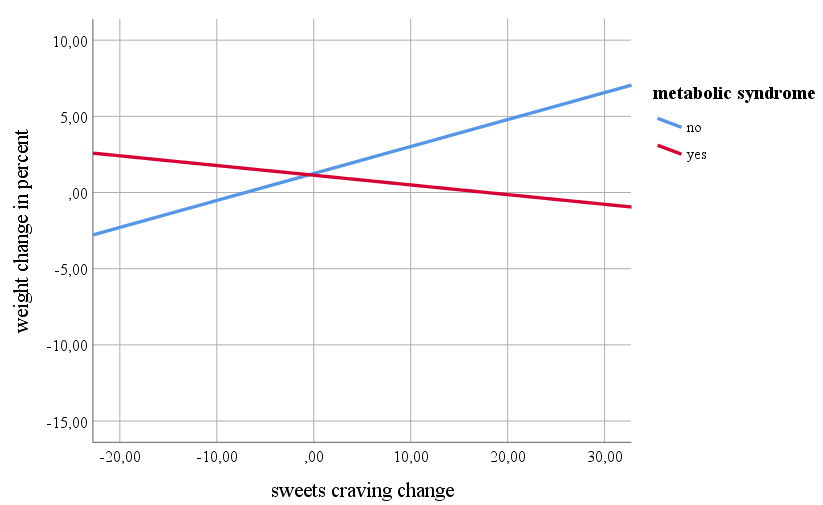


Notes. With increasing sweets craving, patients without metabolic syndrome showed an increase of weight while patients with metabolic syndrome showed a decline of weight. Change of parameters was calculated after four weeks of treatment. Positive values indicate an increase in body weight and sweets craving, negative values indicate a decrease. Metabolic syndrome was defined according to IDF (see [17]) at baseline.
